# Epidemiological impact of public health interventions against diabetes in Qatar: mathematical modeling analyses

**DOI:** 10.3389/fpubh.2023.1167807

**Published:** 2023-06-19

**Authors:** Asalah Alareeki, Susanne F. Awad, Julia A. Critchley, Katie G. El-Nahas, Abdulla O. Al-Hamaq, Salah A. Alyafei, Mohammed H. J. Al-Thani, Laith J. Abu-Raddad

**Affiliations:** ^1^Infectious Diseases Epidemiology Group, Weill Cornell Medical College–Qatar, Cornell University, Doha, Qatar; ^2^World Health Organization Collaborating Centre for Disease Epidemiology Analytics on HIV/AIDS, Sexually Transmitted Infections, and Viral Hepatitis, Weill Cornell Medicine–Qatar, Doha, Qatar; ^3^Department of Population Health Sciences, Weill Cornell Medicine, Cornell University, New York, NY, United States; ^4^Population Health Research Institute, St George's, University of London, London, United Kingdom; ^5^Qatar Diabetes Association, Doha, Qatar; ^6^Public Health Department, Ministry of Public Health, Doha, Qatar; ^7^Department of Public Health, College of Health Sciences, QU Health, Qatar University, Doha, Qatar; ^8^College of Health and Life Sciences, Hamad Bin Khalifa University, Doha, Qatar

**Keywords:** epidemiology, non-communicable disease, risk factors, mathematical modeling, lifestyle management, consumption, legislation, interventions

## Abstract

**Aims:**

To predict the epidemiological impact of specific, and primarily structural public health interventions that address lifestyle, dietary, and commuting behaviors of Qataris as well as subsidies and legislation to reduce type 2 diabetes mellitus (T2DM) burden among Qataris.

**Methods:**

A deterministic population-based mathematical model was used to investigate the impact of public health interventions on the epidemiology of T2DM among Qataris aged 20–79 years, which is the age range typically used by the International Diabetes Federation for adults. The study evaluated the impact of interventions up to 2050, a three-decade time horizon, to allow for the long-term effects of different types of interventions to materialize. The impact of each intervention was evaluated by comparing the predicted T2DM incidence and prevalence with the intervention to a counterfactual scenario without intervention. The model was parameterized using representative data and stratified by sex, age, T2DM risk factors, T2DM status, and intervention status.

**Results:**

All intervention scenarios had an appreciable impact on reducing T2DM incidence and prevalence. A lifestyle management intervention approach, specifically applied to those who are categorized as obese and ≥35 years old, averted 9.5% of new T2DM cases by 2050. An active commuting intervention approach, specifically increasing cycling and walking, averted 8.5% of new T2DM cases by 2050. Enhancing consumption of healthy diets including fruits and vegetables, specifically a workplace intervention involving dietary modifications and an educational intervention, averted 23.2% of new T2DM cases by 2050. A subsidy and legislative intervention approach, implementing subsidies on fruits and vegetables and taxation on sugar-sweetened beverages, averted 7.4% of new T2DM cases by 2050. A least to most optimistic combination of interventions averted 22.8–46.9% of new T2DM cases by 2050, respectively.

**Conclusions:**

Implementing a combination of individual-level and structural public health interventions is critical to prevent T2DM onset and to slow the growing T2DM epidemic in Qatar.

## 1. Introduction

Diabetes mellitus (DM), of which 90% of cases are type 2 diabetes mellitus (T2DM), is one of the most rapidly growing global health challenges (1). Globally, in 2021, 537 million (1 in 10) adults were estimated to be living with DM, and by 2045, this number is projected to increase by 46% to 783 million (1). In 2021, 6.7 million deaths and 966 billion USD in health expenditure were due to DM (1). The Middle East and North Africa (MENA) region has the highest prevalence of DM worldwide at 16.2%, where 73 million adults are living with DM, and by 2045, this number is projected to increase by 87% to 136 million (1).

Qatar, one of MENA countries, has a high prevalence of DM and its key modifiable risk factors, where in 2023, Qataris aged 20–79 years had an estimated prevalence of DM, obesity, physical inactivity, and smoking of 17.8, 53.1, 46.5, and 20.7%, respectively. Approximately 80% of Qataris have at least 2 risk factors for DM (2). Recent epidemiological studies investigating T2DM among Qataris forecasted that the prevalence of T2DM will significantly increase over the next three decades, and that obesity will continue to be the main driver of the T2DM epidemic (3–5).

Such high T2DM burden results in economic and social costs, where national T2DM health expenditure is estimated to account for up to 32% of Qatar's total health expenditure by 2050 (3). With such a pervasive public health challenge, individuals, families, and the wider society experience reduced quality of life, premature loss of workforce, and early mortality due to DM (3).

Tackling DM is a critical priority for policymakers in Qatar, as it is outlined in the National Health Vision 2030 (6). The current public health response has primarily focused on providing quality case management and treatment, as well as raising awareness and promoting behavioral interventions through educational campaigns emphasizing individuals' responsibility to address their risk of developing DM. However, this has been insufficient at reducing the growing burden of T2DM. One of the main challenges of the DM response has been the lack of a comprehensive understanding of DM epidemiology, its incidence, drivers, and potential interventions to tackle this epidemic. Therefore, this study used mathematical modeling to investigate the epidemiological impact of specific, primarily structural public health interventions on T2DM epidemiology among Qataris over three decades. The study was informed by previous research on the impact of generic “what-if” interventions on key T2DM-related modifiable risk factors in Qatar (4). The study found that significant reductions in T2DM incidence could be achieved by reducing obesity, while comparatively modest reductions were observed by reducing physical inactivity and smoking, or by increasing physical activity (4).

This study aimed to investigate the impact of five interventions and their specific scenarios on T2DM prevalence and incidence. The interventions were chosen based on evidence from experimental designs or observational studies in the global literature indicating their potential efficacy/effectiveness in reducing T2DM and on their feasibility and relevance for Qatar's cultural and socio-economic context, which were determined through stakeholder engagement during the early phases of the study. The study adopted a public health approach, focusing on the implementation of select and relevant prevention interventions that target the drivers of T2DM incidence, which have the potential to effectively reduce T2DM incidence and control further epidemic growth in Qatar.

The first intervention investigated a lifestyle management intervention applied to populations at high-risk of T2DM and included three specific scenarios. The second intervention investigated increased use of different modes of active commuting and included two specific scenarios. The third intervention investigated increased consumption of healthy diets, including fruits and vegetables, and included four specific scenarios. The fourth intervention investigated the implementation of a subsidy and legislation intervention and included three specific scenarios. The fifth intervention investigated the impact of implementing the most and least optimistic package of the above four interventions.

## 2. Methods

**Figure 1:**
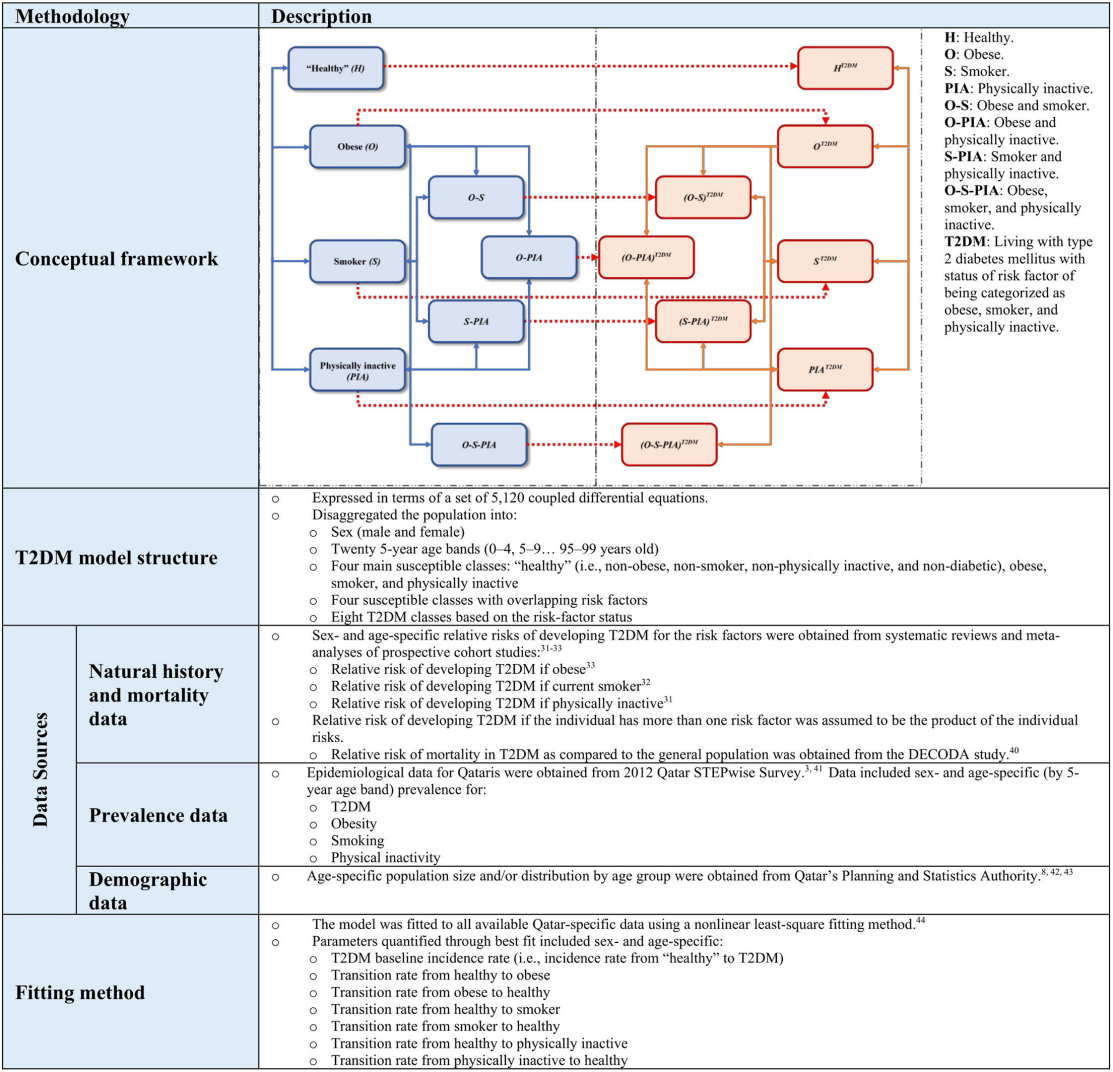
Summary description of the mathematical modeling methodology applied in this study. Further details have been published previously in several applications of this model (3, 4, 7, 8, 10).

In this study, we extended a model developed by Awad et al. to predict the impact of public health interventions on T2DM epidemiology among Qataris aged 20–79 years. This age range follows the convention typically used by the International Diabetes Federation for adults (1). The model structure, assumptions, and parametrization of T2DM natural history, risk factors, and demographics of Qataris are summarized in Supplementary Table 1 and Figure 1. Previous publications provided detailed description of the model, its calibration, results, and figures of the calibration as well as several applications of the model (3, 4, 7–10).

Briefly, this study utilized a deterministic compartmental model expressed in terms of a set of differential equations to predict the impact of specific public health interventions on T2DM epidemiology among Qataris aged 20–79 years. The model stratifies the population by sex, age group (20 age bands), T2DM status, and the presence or absence of three major T2DM-related risk factors and their overlap: obesity, physical inactivity, and smoking. Input parameters and data for the model were obtained from available population-level data and T2DM natural history data. Other parameters were derived through fitting the model to existing epidemiological data on T2DM, T2DM-related risk factors, and demographics of the Qatari population using a nonlinear least-square fitting method (11). This approach allowed for the best fit of the sex- and age-specific epidemiological measures to determine T2DM incidence rate, and transition rates between healthy, obese, smoker, and physically inactive states (Figure 1).

Sex- and age-specific T2DM prevalence and incidence were forecasted using this model for the period from 2021 to 2050. MATLAB 2019a was used to implement the mathematical model and to conduct all analyses. The model was validated by ensuring that it fitted all empirical data related to T2DM in Qatar and that it provided a consistent and coherent picture of T2DM epidemiology in Qatar. Sensitivity and uncertainty analyses were conducted in previous applications of this model, which affirmed its validity and reliability to forecasting Qatar's T2DM epidemic (3–5, 7–10). In this study, we investigated different scenarios for each intervention strategy. These scenarios can be considered as sensitivity analyses of how T2DM epidemiology would change at different levels of the intervention.

### 2.1. Plan of analysis

Five intervention approaches were modeled and investigated among Qataris. Interventions, and their specific scenarios, were selected based on their relevance to Qatar's cultural and socio-economic context, that is those that seemed feasible and relevant in discussion with local diabetes stakeholders including policy makers and public health specialists. The effectiveness of the interventions was parameterized by literature reviews applying evidence from systematic reviews and meta-analyses or high quality randomized controlled trials (12–23). The uptake and adherence levels to the interventions were set at assumed reasonable and realistic values for the Qatari population affected by the intervention.

In the model, the impact of an intervention was assessed by comparing the predicted T2DM prevalence and incidence in the presence of the intervention to a counterfactual scenario with the absence of the intervention. Each intervention was assumed to be initiated in 2021, scaled up to a certain year that varied for each intervention, and then maintained up to 2050. The start year was set at 2021 to provide a prediction time horizon for the simulations of three decades ending in 2050. This allows for the long-term effects of different types of interventions to materialize. Each of these interventions is described in Table 1 and discussed briefly below.

**Table 1:** Summary of the investigated interventions and their scenarios.

**Scenario description**	**Uptake of intervention**	**Adherence to intervention**	**Scale-up time interval**	**Maintenance time interval**	**Intervention assumptions**
**Intervention 1: lifestyle management intervention applied to populations at high-risk of T2DM**					
Scenario 1: lifestyle management programmes applied to those who are categorized as obese	50%	50%	2021–2025	2026–2050	Assumed to reduce the risk of developing T2DM by IRR = 0.70—a reduction of 30% (12, 13). This IRR value is the average of the evidence-based effect sizes of IRR = 0.65 (12) and IRR = 0.74 (13).
Scenario 2: lifestyle management programmes applied to those who are categorized as ≥50 years old	50%	50%	2021–2025	2026–2050	Assumed to reduce the risk of developing T2DM by IRR = 0.70—a reduction of 30% (12, 13). This IRR value is the average of the evidence-based effect sizes of IRR = 0.65 (12) and IRR = 0.74 (13).
Scenario 3: lifestyle management programmes applied to those who are categorized as obese and ≥35 years old	50%	50%	2021–2025	2026–2050	Assumed to reduce the risk of developing T2DM by IRR = 0.70—a reduction of 30% (12, 13). This IRR value is the average of the evidence-based effect sizes of IRR = 0.65 (12) and IRR = 0.74 (13).
**Intervention 2: increasing use of different modes of active commuting**					
Scenario 1: public transportation use	40%	100%	2021–2030	2031–2050	Assumed to reduce BMI by 0.51 kg/m^2^, (15) leading to an obesity prevalence reduction from 53.3 to 50.9% by 2030 (16, 17). The intervention was also assumed to increase the level of physical activity among those physically inactive to reach that of the level of physical activity among the healthy general population (18, 19).
Scenario 2: cycling or walking	20%	100%	2021–2030	2031–2050	Assumed to reduce BMI by 1.68 kg/m^2^ (15), leading to an obesity prevalence reduction from 53.3 to 50.4% by 2030 (16, 17). The intervention was also assumed to increase the level of physical activity among individuals under the intervention whereby T2DM risk is reduced by a RR = 0.76—a reduction of 24%.
**Intervention 3: increasing consumption of healthy diets including fruits and vegetables**					
Scenario 1: consumption of fruits and vegetables	50%	100%	2021–2030	2031–2050	Assumed to reduce the risk of developing T2DM with a RR = 0.93—a reduction of 7% (20).
Scenario 2: consumption of vegetables	50%	100%	2021–2030	2031–2050	Assumed to reduce the risk of developing T2DM with a RR = 0.90—a reduction of 10% (20).
Scenario 3: consumption of green leafy vegetables	50%	100%	2021–2030	2031–2050	Assumed to reduce the risk of developing T2DM with a RR = 0.87—a reduction of 13% (20).
Scenario 4: workplace intervention involving dietary modifications and an educational intervention	50%	100%	2021–2030	2031–2050	Assumed to reduce the risk of developing T2DM with a RR = 0.93—a reduction of 7% (20, 21). Assumed also to reduce BMI by 2.4 kg/m^2^ (22, 23), leading to an obesity prevalence reduction from 53.3 to 34.8% by 2030 (16, 17). The target population is the working adult population aged 20–65 years old.^*^
**Intervention 4: implementing a subsidy and legislation intervention**					
Scenario 1: subsidies on fruits and vegetables	20%	100%	2021–2030	2031–2050	Assumed to reduce BMI by 0.16 kg/m^2^ (22, 23), leading to an obesity prevalence reduction from 53.3 to 52.6% by 2030 (16, 17).
Scenario 2: taxation on SSB	20%	100%	2021–2030	2031–2050	Assumed to reduce BMI by 0.24 kg/m^2^ (22, 23), leading to an obesity prevalence reduction from 53.3 to 52.3% by 2030 (16, 17).
Scenario 3: subsidies on fruits and vegetables and taxation on SSB	20%	100%	2021–2030	2031–2050	Assumed to reduce BMI by 0.40 kg/m^2^, that is the additive effect of the increments of 0.16 kg/m^2^ and 0.24 kg/m^2^ (22, 23), leading to an obesity prevalence reduction from 53.3 to 51.6% by 2030 (16, 17).
**Intervention 5: implementing combinations of interventions**					
Scenario 1: most optimistic combination package of the above four interventions	Uptake of each included individual scenario is included above	Adherence to each included individual scenario is included above	2021–2030	2031–2050	The package includes the most impactful scenario of each intervention: lifestyle management programmes applied to those who are categorized as obese and ≥35 years old, increased walking and cycling, workplace intervention involving dietary modifications and an educational intervention, and implementing subsidies on fruits and vegetables and taxation on SSB.
Scenario 2: least optimistic combination package of the above four interventions	Uptake of each included individual scenario is included above	Adherence to each included individual scenario is included above	2021–2030	2031–2050	The package includes the least impactful scenario of each intervention: lifestyle management programmes applied to those who are categorized as obese, increased public transportation use, increased consumption of fruits and vegetables, and implementing subsidies on fruits and vegetables.

BMI, Body mass index; IRR, Incidence rate ratio; RR, Relative risk; SSB, Sugar-sweetened beverages; T2DM, Type 2 diabetes mellitus.

* The target population for all intervention scenarios is the total adult population aged 20–79 years old. Only intervention 3, scenario 4, targets the working adult population aged 20–65 years old.

#### 2.1.1. Intervention 1: lifestyle management intervention applied to populations at high-risk of T2DM

The lifestyle management intervention approach, resembling the Finnish Diabetes Prevention Study, reduces the risk of developing T2DM through intensive programmes that support individuals at high risk of developing diabetes (e.g., those with pre-diabetes) to make health promoting changes in dietary habits and exercise, resulting in weight loss. This intervention was assumed to target three high-risk Qatari sub-populations to reduce their risk of developing T2DM: those who are categorized as obese, as ≥50 years old, and as both obese and ≥35 years old (Table 1). Selection of these three target groups was informed by applying the Qatari Diabetes Risk Score, which highlighted a subset of the population at high-risk of developing T2DM (10).

A 50% uptake with 50% adherence to the lifestyle management intervention was assumed among each targeted sub-population. This level of uptake was assumed to be scaled up gradually and to reach its targeted level 5 years after onset of this intervention (2021–2025). The uptake was maintained at this level thereafter until 2050. The intervention reduced the risk of developing T2DM with an incidence rate ratio (IRR) = 0.70, that is, a 30% reduction in risk of T2DM onset (12, 13).

#### 2.1.2. Intervention 2: increasing use of different modes of active commuting

The active commuting intervention approach reduces the risk of developing T2DM by lowering population mean body mass index (BMI) and increasing physical activity levels (Table 1). The modeled uptake of active commuting was scaled up gradually to reach its targeted level after a decade (2021–2030). The uptake was maintained at this level thereafter until 2050. Two scenarios were modeled.

In the first scenario, use (uptake) of public transportation among 40% of Qataris was implemented. This intervention was assumed to reduce BMI among the total population by 0.51 kg/m^2^, based on pooled evidence. Applying an established method to transform changes in population mean BMI to changes in obesity prevalence (16, 17), this change in BMI would reduce obesity prevalence among the total population from 53.3 to 50.9% by 2030. The intervention was also assumed to increase the level of physical activity among those physically inactive to reach the level of physical activity among the healthy general population (18, 19).

In the second scenario, cycling or walking by 20% of Qataris was implemented. This intervention was assumed to reduce BMI among the total population by 1.68 kg/m^2^, based on pooled evidence. This change in BMI would reduce obesity prevalence among the total population from 53.3 to 50.4% by 2030. The intervention was also assumed to independently increase the level of physical activity among individuals under the intervention whereby T2DM is reduced by a relative risk (RR) = 0.76, that is, a reduction of 24% in risk of T2DM onset, based on pooled evidence. The latter scenario differs from the first in its physical activity assumption as cycling and walking involves more intense physical activity, and therefore, acts as a protective factor against developing T2DM.

#### 2.1.3. Intervention 3: increasing consumption of healthy diets including fruits and vegetables

The dietary intervention approach reduces the risk of developing T2DM by lowering mean BMI through enhanced nutritional education and environmental dietary modifications, such as in workplaces (Table 1). The modeled uptake of increased consumption was assumed to scale up gradually and to reach its targeted level 10 years after initiation (2021–2030). The uptake was maintained at this level thereafter until 2050. Three scenarios were modeled.

In the first scenario, increased consumption of fruits and vegetables among 50% of Qataris was implemented. The intervention was assumed to reduce the risk of developing T2DM with a RR = 0.93—a 7% reduction in risk of T2DM onset, based on pooled evidence. In the second scenario, increased consumption of vegetables by 50% of Qataris was implemented. The intervention was assumed to reduce the risk of developing T2DM with a RR = 0.90—a reduction of 10% in risk of T2DM onset, based on pooled evidence. In the third scenario, increased consumption of leafy green vegetables among 50% of Qataris was implemented. The intervention was assumed to reduce the risk of developing T2DM with a RR = 0.87—a reduction of 13% in risk of T2DM onset, based on pooled evidence.

In the fourth scenario, a complex workplace dietary intervention involving workplace environmental dietary modifications and an educational intervention, that also increased fruit and vegetable consumption, was implemented among 50% of Qataris of working age, 20–65 years of age, in whom the risk of onset of T2DM is elevated (24). This intervention was modeled informed by the results of the Food Choice at Work cluster controlled trial (23). The intervention was assumed to reduce the risk of developing T2DM with a RR = 0.93—a reduction of 7% in risk of T2DM onset, based on pooled evidence (20, 21). The intervention was also assumed to reduce BMI among individuals under the intervention by 2.4 kg/m^2^, based on factoring the observed change in BMI in the Food Choice at Work trial and extrapolating the effect for a 3-year scale-up (21, 22). This change in BMI would lead to an obesity prevalence reduction strictly among individuals undergoing the intervention from 53.3 to 34.8% by 2030 (16, 17). Admittedly such intervention is somewhat ambitious and aspirational, but it is useful to investigate to provide a context for the size of the impact of such interventions.

#### 2.1.4. Intervention 4: implementing a subsidy and legislation intervention

The subsidy and legislation intervention reduces the risk of developing T2DM indirectly by reducing mean BMI levels through healthier food consumption (Table 1). Informed by the National Institutes of Health's model of weight change, applying legislation on food consumption would lower caloric intake, thereby reducing mean BMI levels, such that 50% of the weight decrease should be reached within 1 year of intervention onset, and 100% of the weight change should be reached within 3 years (22, 23). The modeled uptake of this intervention was to be scaled up gradually to reach its targeted level after a decade (2021–2030). The uptake was maintained at this level thereafter until 2050. Three scenarios were modeled.

In the first scenario, an application of subsidies was assumed to lead to an uptake of 20% in the consumption of healthier foods such as fruits and vegetables. This intervention was assumed to reduce BMI among the total population by 0.16 kg/m^2^, based on pooled evidence (22, 23). This change in BMI would lead to an obesity prevalence reduction among the total population from 53.3 to 52.6% by 2030 (16, 17).

In the second scenario, an application of taxation on sugar-sweetened beverages (SSB) was assumed to lead to an uptake of 20% in reduced consumption of SSB, that is 20% of the population reduced their consumption. This intervention was assumed to reduce BMI among the total population by 0.24 kg/m^2^, based on pooled evidence (22, 23). This change in BMI would lead to an obesity prevalence reduction among the total population from 53.3 to 52.3% by 2030 (16, 17).

In the third scenario, the two scenarios above were applied together assuming their effects are additive. Accordingly, this intervention reduced BMI by 0.40 kg/m^2^, that is, the additive effect of the increments of 0.16 and 0.24 kg/m^2^. This change in BMI would reduce obesity prevalence among the total population from 53.3 to 51.6% by 2030 (16, 17).

#### 2.1.5. Intervention 5: implementing combinations of interventions

Two combination packages of the above interventions were also modeled (Table 1). The first represented the most optimistic scenario of combining the above four interventions by including the most impactful scenario of each intervention. The second represented the least optimistic scenario of combining the above four interventions, including the least impactful scenario of each. Accordingly, these two packages bracket the utility of combining the above four interventions.

## 3. Results

### 3.1. T2DM burden between 2021 and 2050

Calibration of the model to the Qatari population and its epidemiological data has been published previously. T2DM prevalence among 20–79-year-old Qataris, the age group to which the interventions were applied, was projected by the model to increase from 17.1% in 2021 to 29.5% in 2050 (Supplementary Figure 1). The prevalent number of T2DM cases is expected to increase from 33,821 in 2021 to 84,516 in 2050. The annual number of new (incident) T2DM cases is expected to increase from 2,145 in 2021 to 3,931 in 2050.

### 3.2. Impact of lifestyle management intervention applied to populations at high-risk of T2DM

**Figure 2:**
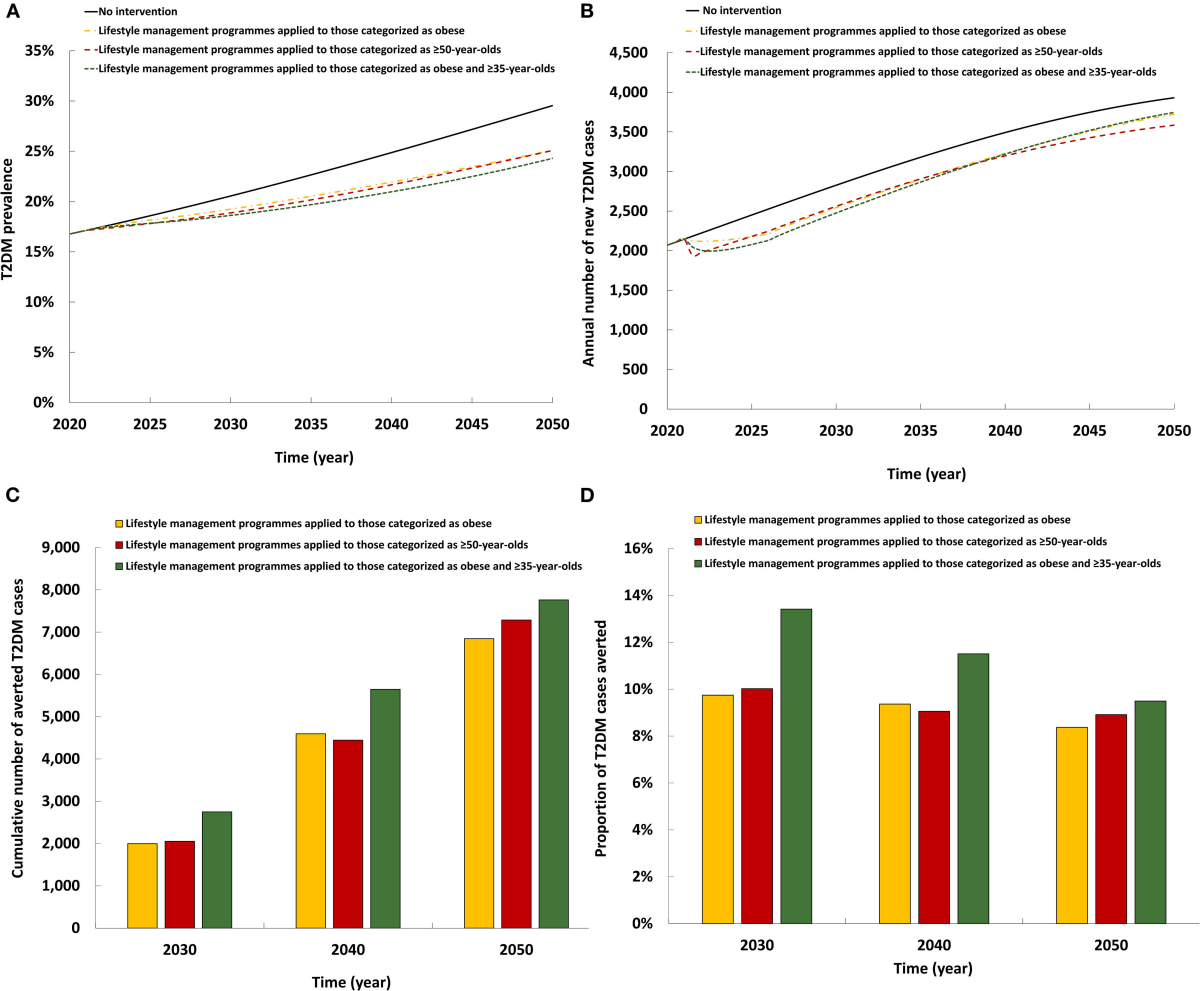
Projected impact of the lifestyle management intervention on **(A)** T2DM prevalence, **(B)** annual number of new T2DM cases, **(C)** cumulative number of averted T2DM cases by this intervention, and **(D)** proportion of T2DM cases averted by this intervention.

The three modeled scenarios of the lifestyle management intervention had roughly comparable impact (Figure 2). All three reduced T2DM prevalence in 2050 by 4.4–5.2 absolute percentage points and reduced the annual number of new T2DM cases by 4.7–8.8%. By 2050, the cumulative number of averted T2DM cases by the intervention ranged between 6,845 and 7,762. Also, by 2050, the proportion of T2DM cases averted by the intervention ranged between 8.4 and 9.5%. Lifestyle management programmes when applied to individuals categorized as both obese and ≥35 years old had a slightly higher impact on T2DM prevalence and incidence than when applied to individuals categorized as obese or as ≥50 years old.

### 3.3. Impact of increasing use of different modes of active commuting

**Figure 3:**
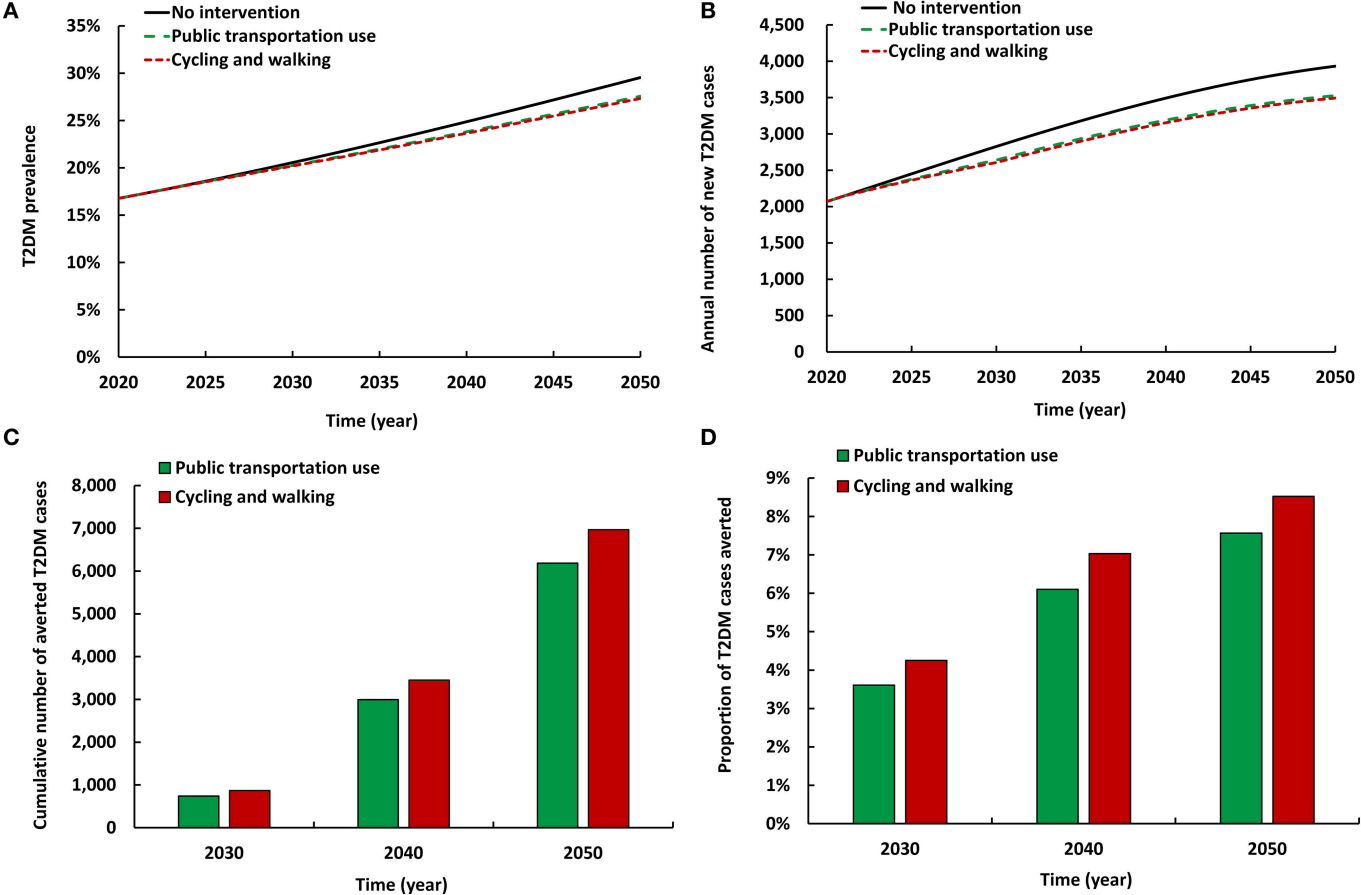
Projected impact of increasing use of different modes of active commuting on **(A)** T2DM prevalence, **(B)** annual number of new T2DM cases, **(C)** cumulative number of averted T2DM cases by these interventions, and **(D)** proportion of T2DM cases averted by these interventions.

The two modeled scenarios of active commuting intervention had comparable impact (Figure 3). Both reduced T2DM prevalence in 2050 by 1.9–2.2 absolute percentage points and reduced the annual number of new T2DM cases by 10.3–11.1%. By 2050, the cumulative number of averted T2DM cases by the intervention ranged between 6,187 and 6,970. Also, by 2050, the proportion of T2DM cases averted by the intervention ranged between 7.6 and 8.5%. Increased cycling and walking had a slightly higher impact on T2DM prevalence and incidence than increased use of public transportation (please note intervention coverage in each scenario is different; Table 1).

### 3.4. Impact of increasing consumption of healthy diets including fruits and vegetables

**Figure 4:**
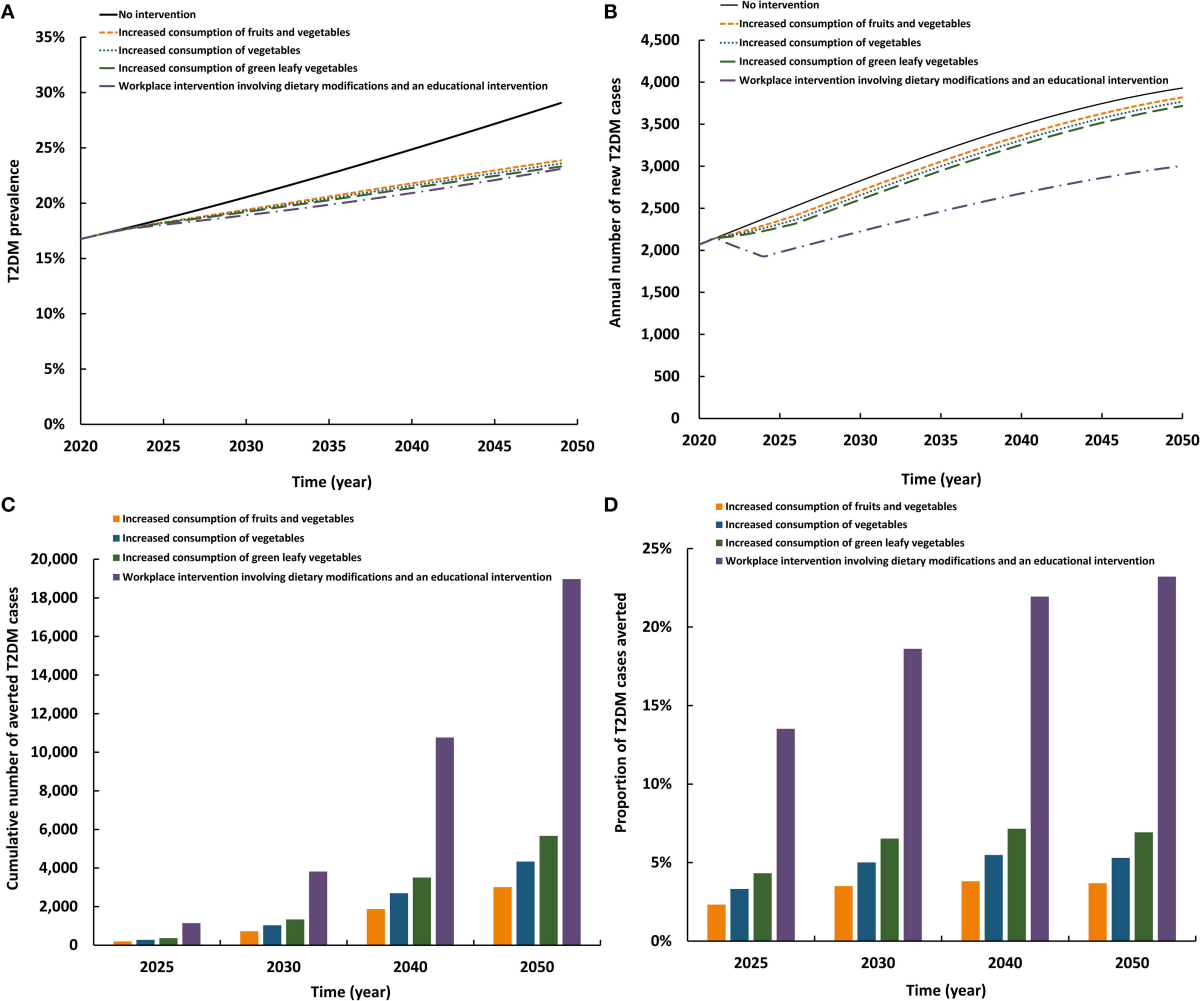
Projected impact of increasing consumption of healthy diets including fruits and vegetables on **(A)** T2DM prevalence, **(B)** annual number of new T2DM cases, **(C)** cumulative number of averted T2DM cases by this intervention, and **(D)** proportion of T2DM cases averted by this intervention.

The first three scenarios for increasing fruit and vegetable consumption had roughly comparable impact, whereas the fourth scenario had a considerably higher impact (Figure 4). The four scenarios reduced T2DM prevalence in 2050 by 5.4–6.1 absolute percentage points and reduced the annual number of new T2DM cases by 2.8–23.5%. By 2050, the cumulative number of averted T2DM cases by the intervention ranged between 3,009 and 18,975. Also, by 2050, the proportion of T2DM cases averted by the intervention ranged between 3.7 and 23.2%. Implementing a complex workplace dietary and environmental intervention (that includes increased fruit and vegetable consumption) had a substantially higher impact than increased consumption of fruits and vegetables, of vegetables, or of leafy greens on their own.

### 3.5. Impact of implementing a subsidy and legislation intervention

**Figure 5:**
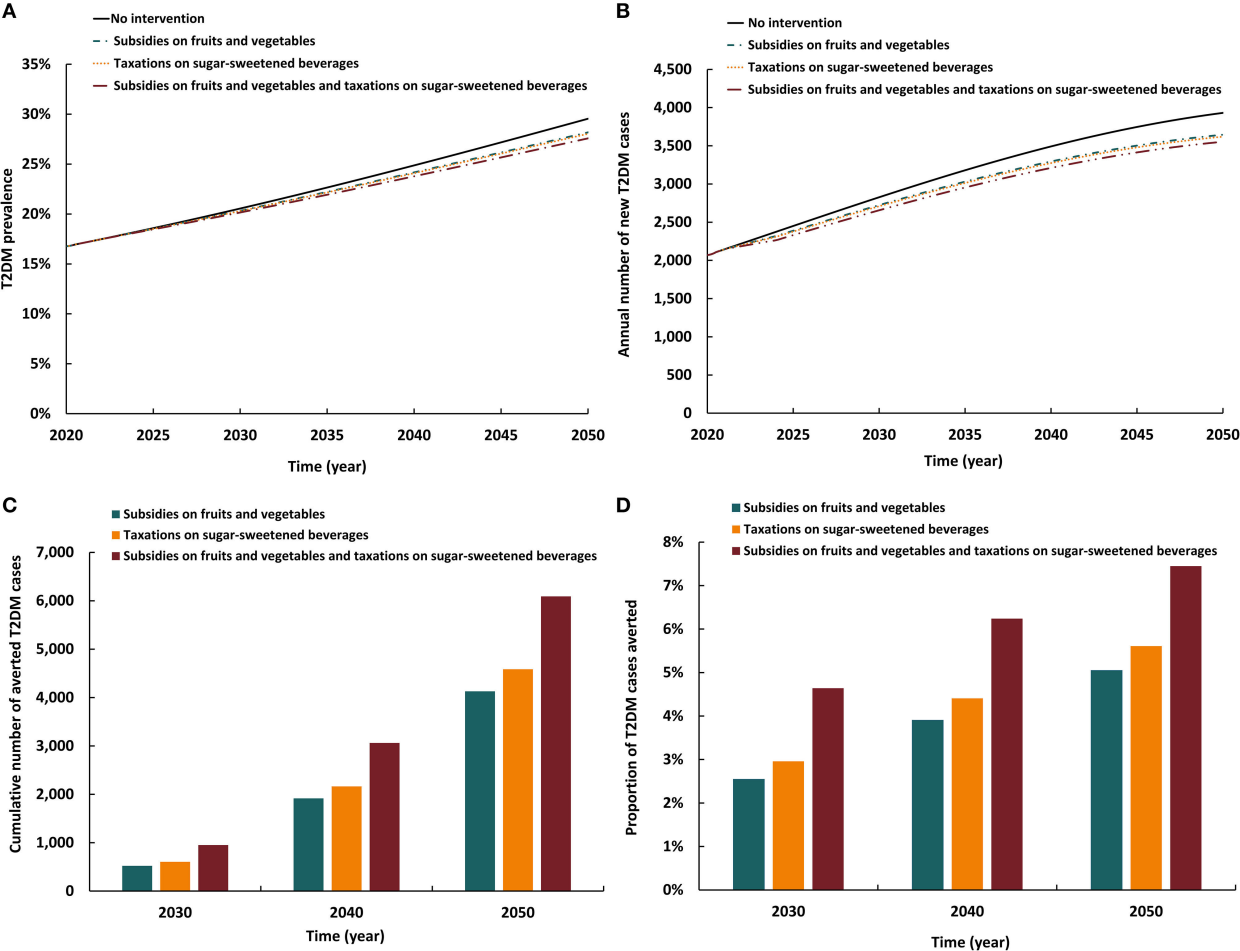
Projected impact of implementing a subsidy and legislation intervention on **(A)** T2DM prevalence, **(B)** annual number of new T2DM cases, **(C)** cumulative number of averted T2DM cases by this intervention, and **(D)** proportion of T2DM cases averted by this intervention.

The three modeled interventions involving subsidy and legislation had similar impacts (Figure 5). The three reduced T2DM prevalence in 2050 by 1.3–1.9 absolute percentage points and the annual number of new T2DM cases by 7.3–9.6%. By 2050, the cumulative number of averted T2DM cases by the intervention ranged between 4,130 and 6,090. Also, by 2050, the proportion of T2DM cases averted by the intervention ranged between 5.1 and 7.4%. The scenario combining subsidies on fruits and vegetables with taxation on SSB had the highest impact on T2DM prevalence and incidence than either intervention scenarios alone.

### 3.6. Impact of implementing subsidy and legislation intervention

**Figure 6:**
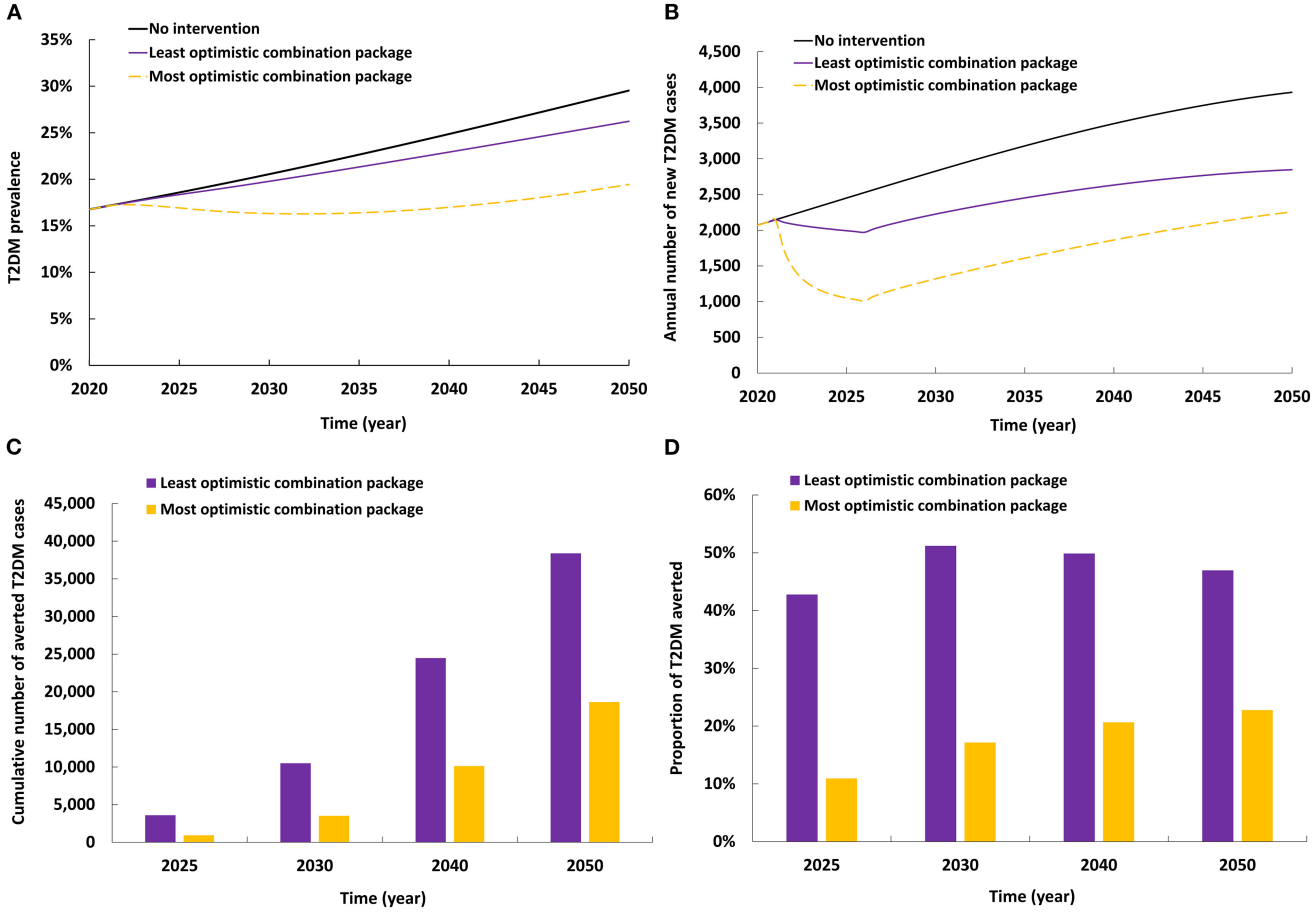
Projected impact of implementing combinations of interventions on **(A)** T2DM prevalence, **(B)** annual number of new T2DM cases, **(C)** cumulative number of averted T2DM cases by combinations of interventions, and **(D)** proportion of T2DM cases averted by combinations of interventions.

The impact of combining packages of interventions is shown in Figure 6. The least optimistic combination of interventions reduced T2DM prevalence in 2050 by 3.3% and reduced the annual number of new T2DM cases by 27.6%. By 2050, 18,619 new T2DM cases (22.8%) were averted by this package of interventions.

The most optimistic combination of interventions reduced T2DM prevalence in 2050 by 10.1% and reduced the annual number of new T2DM cases by 42.6%. By 2050, 38,379 new T2DM cases (46.9%) were averted by this package of interventions.

## 4. Discussion

T2DM is the leading public health challenge in Qatar. This study investigated the epidemiological impact of specific, primarily structural public health interventions at reducing T2DM burden. Each intervention approach and its scenarios reduced T2DM incidence appreciably. However, combinations of these interventions were most effective and could potentially avert between 23 and 47% of new T2DM cases. These findings demonstrate the difficulty of controlling the T2DM epidemic using a single approach, as no single public health intervention had a major reduction on the projected T2DM prevalence and incidence throughout the three decades of investigation. Tackling this epidemic will require major investments in specific, large-scale interventions applied together. Although we did not analyze this directly, other evidence suggests such approaches may also be more equitable e.g., by sex and socio-economic status (9).

While all interventions had an impact, there were differences in the immediacy of the impact. Intervention approaches that directly affected onset of T2DM, that is lifestyle management programmes applied to populations at high-risk as well as increasing consumption of fruits and vegetables, had an immediate impact on reducing T2DM incidence during the scale-up time of the intervention, but this reduction saturated by end of scale-up. Interventions that affected onset of T2DM indirectly by affecting obesity and physical inactivity, i.e., increasing active commuting and subsidy and legislative interventions, had limited impact in the short term, but large long-term impact, with benefits increasing over time, even well after end of scale-up.

Interventions that were applied to the age group in which T2DM onset is rising rapidly, those 40–55 years of age (24), averted more T2DM cases, as the intervention directly affected those that are likely to progress to T2DM within the next few years. This was the case for the scenario implementing a complex workplace dietary intervention that had a substantial reduction in T2DM incidence as it was applied to the age group that experiences the highest rate of T2DM onset (24), thereby delaying onset of T2DM to older age or preventing it altogether.

Impact of the interventions on reducing T2DM incidence was higher than on reducing T2DM prevalence. This outcome is due to the already existing high prevalence of T2DM in Qatar and to the nature of the interventions consisting of public health strategies that aim specifically to prevent onset of T2DM. Some scenarios did not have a large impact on T2DM burden, which was partly due to the limited intervention adoption/coverage assumed. For some scenarios, we assumed a realistic adoption of only 20% if the strategy were implemented in Qatar. A higher impact would be observed had we assumed a larger uptake.

Although the response to T2DM in Qatar is evolving, it lags behind the growing epidemic and most prevention and health promotion efforts remain small-scale, generic, and didactic (25). Current public health response focuses on behavioral and lifestyle interventions through education and awareness campaigns. There remains a perception, among policy makers worldwide, that it is an individual responsibility to address the risk of developing T2DM. Resources have been allocated and spent to raise awareness about T2DM, the importance of T2DM screening, and treatment options. Although such T2DM response has had an impact on controlling glucose levels in the population and on averting T2DM complications, i.e., tertiary prevention, such efforts are insufficient in creating a major impact on T2DM epidemiology and controlling T2DM incidence. In line with Qatar's National Diabetes Strategy (2016–2022), structural and more upstream population-level interventions are needed, i.e., primary prevention.

While structural population-based strategies have the potential to be effective in reducing the burden of T2DM (26–28), their implementation is often hindered by various barriers and challenges, such as political, policy, and social constraints (29). These interventions are more difficult to measure and assess their tangible outcomes in both the short- and long-term, particularly in experimental designs with population-level randomization (29). However, this study provides quantitative evidence advocating for the implementation of such interventions by demonstrating their potential epidemiological impact over the next three decades.

The uptake and adherence levels assumed in this study's scenarios remains to be validated in actual application, given Qatar's unique socio-cultural and socio-economic context, which is rapidly evolving. To address this challenge, pilot interventions should be conducted to test the feasibility of the proposed interventions and adjust uptake levels accordingly. This study emphasizes the importance of adopting a public health approach that intervenes on whole populations, as it can effectively tackle the burden of T2DM by addressing the fundamental drivers of DM incidence and by being more cost-effective in the long run (26–28). Policy-makers and public health specialists should prioritize resource allocation to structural population-based strategies to reduce the burden of T2DM and other chronic diseases.

The present study is grounded on the conceptual framing of taking a public health approach to tackle the growing T2DM epidemic with the aim of improving quality, effectiveness, and cost-effectiveness of health interventions. This approach considers T2DM epidemiology as reflecting a “sick population” rather than “sick individuals.” The current public health approach focused on “sick individuals” will have limited success in stemming the rising tide of T2DM incidence and prevalence, as it does not address the drivers of T2DM incidence. Meanwhile, an approach focused on a “sick population” seeks to shift the distribution of T2DM drivers and underlying risk factors (i.e., obesity) so that disease onset is prevented in the first place. Our findings advocate for the relevance and effectiveness of this approach.

The evidence presented here demonstrates that policy level facilitation is necessary to create an environment that makes the “healthier choice the easier choice.” Structural public health interventions have the greatest potential to substantially impact the T2DM epidemic. Such structural interventions are fiscal, legislative, or environmental in nature, and outside an individual's control. They include policies to reduce consumption of unhealthy foods through fiscal policies, e.g., increasing taxation on sugar-sweetened beverages, or providing subsidies for healthier foods, and increasing consumption of fruits and vegetables. Other interventions include changes to food environments, e.g., altering the types of foods in vending machines located in schools, workplaces, or other community settings. Increases in active commuting could be achieved through increased taxation on roads, cars, fuel, or car parking charges, alongside improved cycling and walking routes and infrastructure. These contrast with, but complement, behavioral interventions applied to individuals at risk for T2DM with the aim of changing their diets and/or increasing their physical activity levels.

This study has limitations. The model was parameterized using global data for effect sizes of interventions, effects of risk factors on T2DM, and effects of T2DM on mortality, but the representativeness of these effect sizes for the Qatari population remains unknown. However, these effect sizes are based on pooled estimates from multiple settings worldwide (12, 13, 15–23, 30–32), thereby perhaps accounting for some variability in the global population. Effect sizes should also represent biological mechanisms that may tend to be universal in their effect.

Data used to parameterize the model for physical inactivity levels were self-reported and potentially inflated relative to physical inactivity levels assessed using objective biomarkers. In analyses that included increasing physical activity levels, we only factored the direct effect of physical activity on T2DM incidence, which may underestimate its impact, given that physical activity may also indirectly impact T2DM incidence by reducing obesity. However, most studies have identified only small effects of physical activity on weight change. We strictly focused on reducing obesity in the population, but this may underestimate the impact of incremental reductions in BMI on T2DM incidence, i.e., BMI reductions that do not cross the obesity threshold, but can still reduce T2DM incidence (14, 33, 34). BMI ≥30 kg/m^2^ was used to define obesity as it is a pragmatic measure often used in surveys (35); however, this may not be the best measure to capture the impact of obesity on T2DM incidence (as opposed to say abdominal obesity) (35).

Albeit we used current and relevant epidemiological data to parameterize our model, the availability of more nationally representative surveys with T2DM data could have improved its accuracy in long-term predictions. Nonetheless, previous applications of the model included sensitivity and multivariate uncertainty analyses, which confirmed its validity and reliability in making predictions (3, 4, 7–10). The sensitivity analyses showed that the model's outcomes were highly dependent on the RR of developing T2DM when obese, as expected since obesity is the leading cause of T2DM in Qatar. Additionally, the results indicated that the self-reported rates of physical inactivity impacted (to a modest extent) the model's accuracy, suggesting the need for objective biomarkers in physical activity surveys. The multivariate uncertainty analyses indicated narrow uncertainty intervals around the point estimates of the model output, after factoring the uncertainty in input parameters.

Although we focused on the Qatari population due to the availability of nationally representative data, the health benefits of the interventions investigated in this study extend to the expatriate population living in Qatar. Once representative data for the expatriate population become available, the benefits of the interventions should be quantified. Although this study focused on T2DM, the intervention approaches investigated could simultaneously lower the incidence of other serious morbidities such as cardiovascular diseases and cancer. Therefore, the impact estimates should be considered as conservative estimates of the broader health benefits of these public health interventions. The innovative modeling methodology used in this study can be extended beyond Qatar to other countries in the MENA region and elsewhere, particularly those affected by a similar T2DM epidemiology. The findings, indicating the effectiveness of implementing combinations of individual-level and structural interventions, can inform DM response in various countries and could be applicable, to a certain extent, to other countries.

### 4.1. Conclusion

The study findings highlight the critical need to implement a combination of individual-level and structural public health interventions to prevent T2DM onset and slow the growing T2DM epidemic in Qatar. The evidence presented demonstrates that policy level facilitation is necessary to create an environment that makes the “healthier choice the easier choice,” and consequently, to reduce T2DM risk by reducing its key risk factors. Structural public health interventions targeting T2DM and its risk factors have the greatest potential to affect diabetes epidemiology and to slow the epidemic; individually focused interventions targeting those at higher risk cannot have a major impact alone. These structural interventions complement behavioral interventions that are applied to individuals at risk of T2DM with the aim of changing their diets and/or increasing their physical activity.

## Data availability statement

The original contributions presented in the study are included in the article/Supplementary material, further inquiries can be directed to the corresponding author.

## Author contributions

LA-R conceived the study. SFA, JC, and LA-R designed the model and intervention scenarios. SFA and AA conducted the analyses. AA, SFA, and LA-R analyzed and interpreted the results. AA with LA-R wrote the first draft of the manuscript. All authors contributed significantly to this study, interpretation of the results, writing of the manuscript, and approved the final manuscript.
