# Supplementary material for: Epidemiological impact of public health interventions against diabetes in Qatar: mathematical modeling analyses
Source: Front Public Health. 2023 Jun 19;11:1167807. doi: 10.3389/fpubh.2023.1167807 (PMC10315912; doi:10.3389/fpubh.2023.1167807)
Supplement: Supplementary file 1 [file Data_Sheet_1.docx]

Supplementary Material

Epidemiological impact of public health interventions against diabetes in Qatar: mathematical modeling analyses

**Asalah Alareeki,^1,2^ Susanne F. Awad,^1,2,3^ Julia A. Critchley,^4^ Katie G. El-Nahas,^5^ Abdulla O. Al-Hamaq,^5^ Salah A. Alyafei,^6^ Mohammed H. J. Al-Thani,^6^ and Laith J. Abu-Raddad^1,2,3,7,8*^**

^1^Infectious Diseases Epidemiology Group, Weill Cornell Medical College – Qatar, Cornell University, Doha, Qatar

^2^World Health Organization Collaborating Centre for Disease Epidemiology Analytics on HIV/AIDS, Sexually Transmitted Infections, and Viral Hepatitis, Weill Cornell Medicine – Qatar, Doha, Qatar

^3^Department of Population Health Sciences, Weill Cornell Medicine, Cornell University, New York, USA

^4^Population Health Research Institute, St George’s, University of London, London, UK

^5^Qatar Diabetes Association, Doha, Qatar

^6^Public Health Department, Ministry of Public Health, Doha, Qatar

^7^Department of Public Health, College of Health Sciences, QU Health, Qatar University, Doha, Qatar

^8^College of Health and Life Sciences, Hamad bin Khalifa University, Doha, Qatar

***Correspondence:** Professor Laith J. Abu-Raddad, Infectious Disease Epidemiology Group, World Health Organization Collaborating Centre for Disease Epidemiology Analytics on HIV/AIDS, Sexually Transmitted Infections, and Viral Hepatitis, Weill Cornell Medicine - Qatar, Qatar Foundation - Education City, P.O. Box 24144, Doha, Qatar. Telephone: +(974) 4492-8321. Fax: +(974) 4492-8333. E-mail: [lja2002@qatar-med.cornell.edu](mailto:lja2002@qatar-med.cornell.edu).

# Table S1. The model’s assumptions in terms of parameter values.

| ***Assumption*** | ***Age group*** | ***Parameter value (95% CI)*** | | ***Reference*** |
| --- | --- | --- | --- | --- |
|  |  | **Male** | **Female** |  |
| Number of age compartments in the model (each for 5 years; *a*) | - | 20 | 20 | - |
| Relative risk of developing T2DM if obese $\left(RR_{O}\right)$ | All | 6.48 (5.17–8.13) | 8.38 (5.46–12.85) | [^29^](#_ENREF_29) |
| Relative risk of developing T2DM if current smoker $\left(RR_{S}\right)$ | All | 1.42 (1.34–1.50) | 1.33 (1.26–1.41) | [^30^](#_ENREF_30) |
| Relative risk of developing T2DM if physically inactive $\left(RR_{F}\right)$ | 15–69  70–79  ≥80 | 1.45 (1.37–1.54)  1.32 (1.25–1.40)  1.20 (1.14–1.28) | 1.45 (1.37–1.54)  1.32 (1.25–1.40)  1.20 (1.14–1.28) | [^31^](#_ENREF_31) |
| Relative risk of developing T2DM if obese and smoker $\left(RR_{OS}\right)$ | All | 9.20 (6.93–12.20) | 11.15 (6.88–18.12) | Calculated based on[^29^](#_ENREF_29)^,^[^30^](#_ENREF_30) |
| Relative risk of developing T2DM if obese and physically inactive $\left(RR_{OF}\right)$ | 15–69  70–79  ≥80 | 9.40 (7.08–12.52)  8.55 (6.46–11.38)  7.78 (5.89–10.41) | 12.15 (7.48–19.79)  11.06 (6.83–18.12)  10.06 (6.22–16.45) | Calculated based on[^29^](#_ENREF_29)^,^[^31^](#_ENREF_31) |
| Relative risk of developing T2DM if smoker and physically inactive $\left(RR_{SF}\right)$ | 15–69  70–79  ≥80 | 2.06 (1.84–2.37)  1.87 (1.68–2.17)  1.70 (1.53–1.97) | 1.93(1.73–2.17)  1.76 (1.58–1.99)  1.60 (1.44–1.80) | Calculated based on[^30^](#_ENREF_30)^,^[^31^](#_ENREF_31) |
| Relative risk of developing T2DM if obese, smoker, and physically inactive $\left(RR_{OSF}\right)$ | 15–69  70–79  ≥80 | 13.34 (9.49–19.28)  12.15 (8.66–17.65)  11.04 (7.90–16.03) | 16.16 (9.43–27.90)  14.71 (8.60–25.55)  13.37 (7.84–23.19) | Calculated based on[^29-31^](#_ENREF_29) |
| RR of mortality in T2DM as compared to the general population $\left(RR_{M}\right)$ | 20–29  30–39  40–49  50–59  60–69  70–79+ | 3.70  3.30  1.95  1.65  1.62  1.40 | 5.95  5.61  3.41  2.73  2.08  1.78 | [^1^](#_ENREF_1)^,^[^34^](#_ENREF_34) |

Abbreviations: T2DM = Type 2 diabetes mellitus.

# Figure S1. Projected evolution of the T2DM epidemic among Qataris between 2020-2050. A) T2DM prevalence. B) Number of people living with T2DM. C) Annual number of new T2DM cases.


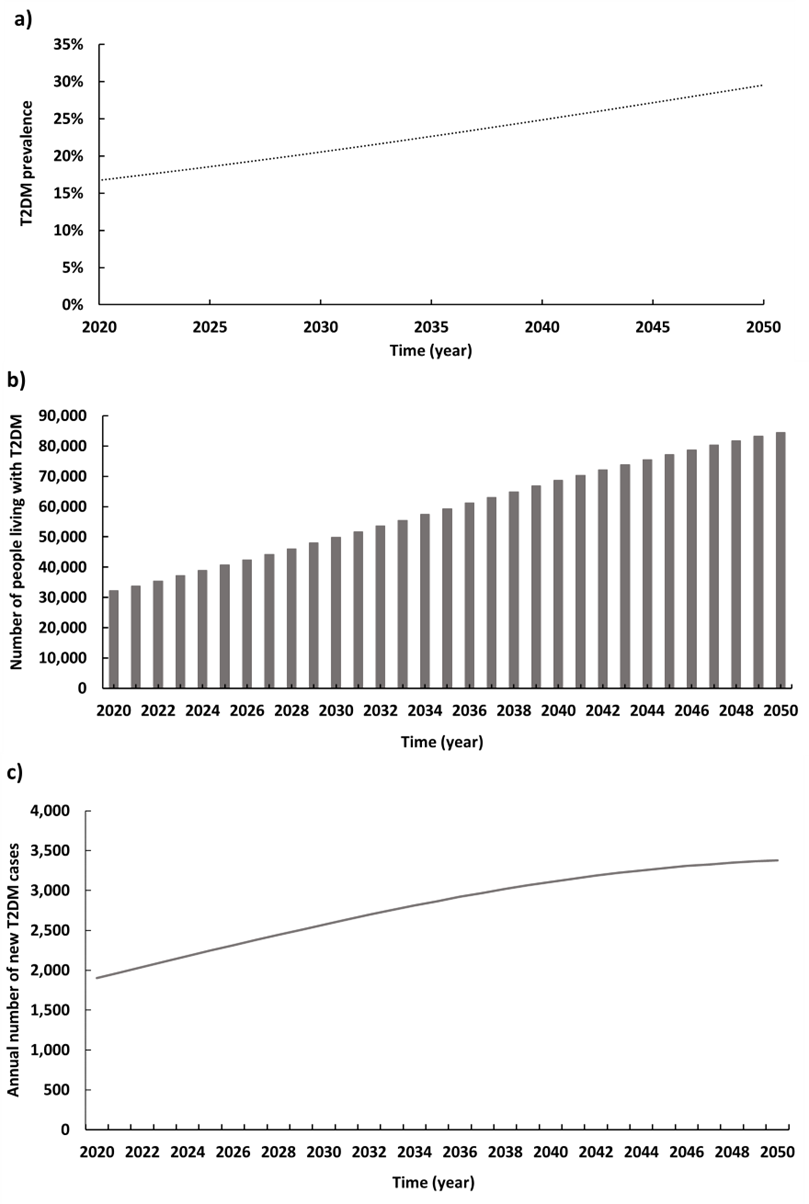


**
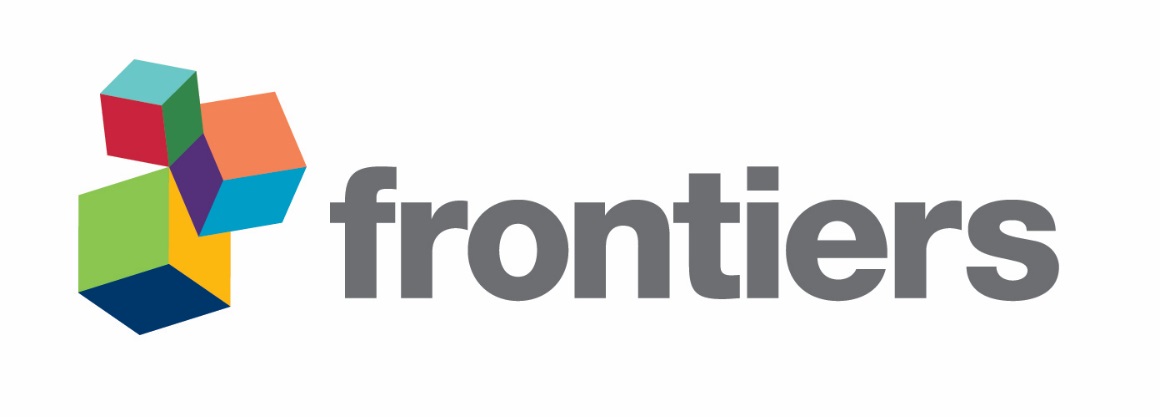
**
